# Local Injections of Superoxide Dismutase Attenuate the Exercise Pressor Reflex in Rats with Femoral Artery Occlusion

**DOI:** 10.3389/fphys.2018.00039

**Published:** 2018-02-02

**Authors:** Jihong Xing, Jian Lu, Jiahao Liu, Jianhua Li

**Affiliations:** ^1^Pennsylvania State Heart and Vascular Institute, The Pennsylvania State University College of Medicine, Hershey, PA, United States; ^2^Department of Emergency Medicine, The First Hospital of Jilin University, Changchun, China

**Keywords:** muscle contraction, blood pressure, peripheral arterial disease, superoxide dismutase, muscle ischemia, oxidative stress

## Abstract

The exercise pressor reflex is amplified in patients with peripheral artery disease (PAD) and in an experimental PAD model of rats induced by femoral artery occlusion. Heightened blood pressure worsens the restricted blood flow directed to the limbs in this disease. The purpose of this study was to determine the role played by muscle oxidative stress in regulating the augmented pressor response to static exercise in PAD. We hypothesized that limb ischemia impairs muscle superoxide dismutase (SOD) thereby leading to abnormal autonomic responsiveness observed in PAD animals, and a chronic compensation of SOD for anti-oxidation improves the exaggerated exercise pressor reflex. Our data show that femoral occlusion decreased the protein levels of SOD in ischemic muscle as compared with control muscle. Downregulation of SOD appeared to a greater degree in the oxidative (red) muscle than in the glycolytic (white) muscle under the condition of muscle ischemia. In addition, the exercise pressor response was assessed during electrically induced static contraction. The data demonstrates that the enhancement of the exercise pressor reflex was significantly attenuated after tempol (a mimetic of SOD, 30 mg over a period of 72 h) was administered into the occluded hindlimb. In the occluded rats, mean arterial pressure (MAP) response was 26 ± 3 mmHg with no tempol and 12 ± 2 mmHg with tempol application (*P* < 0.05 vs. group with no tempol; *n* = 6 in each group). There were no differences in muscle tension development (time-tension index: 12.1 ± 1.2 kgs with no tempol and 13.5 ± 1.1 kgs with tempol; *P* > 0.05 between groups). In conclusion, SOD is lessened in the ischemic muscles and supplement of SOD improves the amplified exercise pressor reflex, which is likely beneficial to the restricted blood flow to the limbs in PAD.

## Introduction

During exercise, sympathetic nervous activity (SNA) increases and this leads to rises in blood pressure (BP) and heart rate (HR), myocardial contractility and peripheral vasoconstriction (Victor et al., 1988; Sinoway et al., 1989). A basic mechanism termed the “Exercise Pressor Reflex” (Coote et al., 1971; McCloskey and Mitchell, 1972; Mitchell et al., 1983; Kaufman and Forster, 1996) is thought to contribute to sympathetic engagement during exercise. This autonomic reflex is initiated as thin fiber afferents arising from contracting skeletal muscle are engaged (McCloskey and Mitchell, 1972; Mitchell et al., 1983; Kaufman and Forster, 1996). This system responds to mechanical deformation of the muscle afferents receptive field as well as to muscle metabolic by-products (Kaufman and Forster, 1996). Group III afferents are predominantly mechanically sensitive (mechanoreceptor) and Group IV afferents are predominantly metabosensitive (metaboreceptor) (Kaufman et al., 1984). When these receptors are stimulated, thin fiber muscle afferent nerves are engaged, cardiovascular nuclei in the brainstem are activated, SNA increases and BP and HR rise (Mitchell et al., 1983).

Peripheral arterial disease (PAD) is atherosclerotic disease with a decrease in blood flow to the arteries of the lower extremities. In this disease, the most common symptom is intermittent claudication, which is worsened by intense exercise due to muscle ischemia but subsides at rest when the metabolic demand of the active muscles is low (Rejeski et al., 2008). Systolic and diastolic BP rise to a greater degree in the PAD patients than in the normal subjects during walking (Baccelli et al., 1997). Furthermore, the exercise pressor reflex plays a role in evoking the exaggerated BP response to walking in PAD patients (Baccelli et al., 1999). In a rat model of femoral occlusion, the SNA and BP responses are also amplified by muscle contraction and stimulation of muscle metabolic receptors (Tsuchimochi et al., 2010; Li and Xing, 2012).

Oxidative stress (OS) plays an important role in the onset and progression of atherosclerosis (Harrison et al., 2003). In PAD, reduced endothelial function due to plaque build-up also stimulates superoxide production (Brevetti et al., 2003; Harrison et al., 2003). Endothelial cells and vascular smooth muscle cells can produce reactive oxygen species (ROS) and in PAD this state of vascular abnormalities with increased OS is coupled with a reduced antioxidant system (Langlois et al., 2001; Fisher-Wellman et al., 2009). This process likely leads to worsening of disease, which may be further exacerbated during exercise due to increases of ROS production (Karamouzis et al., 2004; Rietjens et al., 2007).

The production of superoxide (i.e., ${\text{O}}_{2}^{-}$) is increased in contracting muscles of anesthetized cats and rats (O'Neill et al., 1996) and in the muscles of rats performing intense treadmill exercise (Davies et al., 1982; Zhao et al., 2006). The role played by ${\text{O}}_{2}^{-}$ in generating the exercise pressor reflex is controversial in healthy animals. However, in animals with heart failure and PAD, ROS is likely to play a role in modulating the exercise pressor reflex. It has been reported that the BP response to static muscle contraction was enhanced via an OS mechanism in rat models of heart failure and PAD (Koba et al., 2009; Harms et al., 2017). Of note, among the metabolic receptors engaged in the amplified exercise pressor reflex in PAD, transient receptor potential channel A1 (TRPA1) is responding to ROS (Bandell et al., 2004; Trevisani et al., 2007; Bessac et al., 2008; Kim et al., 2016). ROS are considered as endogenously generated molecule mediators during oxidative stress and/or inflammation (Bandell et al., 2004; Trevisani et al., 2007; Bessac et al., 2008; Kim et al., 2016). Our prior studies further indicate that TRPA1 is engaged in the augmented pressor response to static exercise in occluded rats (Xing et al., 2015; Xing and Li, 2017). Thus, it was postulated that ROS would be a part of the mechanisms leading to the exaggerated exercise pressor reflex in PAD.

Superoxide dismutases (SOD) are a class of enzymes to catalyze the dismutation of superoxide into oxygen and hydrogen peroxide, and considered as an important antioxidant linked to exercise in diseases (Fisher-Wellman et al., 2009). Thus, in this report, one of the purposes was to examine the levels of SOD within oxidative (red) and glycolytic (white) muscles of the occluded hindlimb. We hypothesized that himblimb ischemia impairs muscle SOD thereby leading to abnormal autonomic responsiveness observed in rats with femoral occlusion.

In addition, tiron (a mimetic of SOD) was observed to attenuate the exercise pressor reflex in occluded rats after it was given acutely into arterial blood supply of the hindlimb muscles (Harms et al., 2017). Another study showed that tempol (another SOD mimetic), but not tiron had effects on the reflex in occluded rats (McCord et al., 2011). The difference was likely due to the approaches and periods to administer the antioxidants. Nonetheless, if a chronic compensation of antioxidants following femoral occlusion can attenuate the amplified exercise pressor reflex was unknown. Thus, in this report, we examined the effects of tempol on the exercise pressor reflex as it was locally administered into the occluded msucles over a period of 72 h before the reflex was evoked. We hypothesized that a chronic administration of tempol attenuates the amplified exercise pressor reflex if muscle SOD is impaired in occluded animals.

## Materials and methods

### Animals

All animal experimental procedures were approved by the Institutional Animal Care and Use Committee of Pennsylvania State College of Medicine and complied with the National Institutes of Health (NIH) guidelines.

### Ligation of the femoral artery

Forty-one male Sprague-Dawley rats (250–300 g) were anesthetized with an isoflurane-oxygen mixture (2–5% isoflurane in 100% oxygen). For the western blotting and immunofluorescence experiments, the femoral artery on one limb was surgically exposed, dissected, and ligated ~3 mm distal to the inguinal ligament as previously described (Lu et al., 2013; Xing et al., 2015). In control, the same procedures were performed on the other limb except that a suture was placed below the femoral artery but was not tied. The limbs in which the femoral artery was ligated served as “occluded limbs;” and the other limbs served as “control limbs.” Six rats were used for each time course: 6, 24, and 72 h after the ligation surgery. For the experiment of BP recording, the rats were divided between those that had the right femoral artery ligation (“occluded rats”, *n* = 12) and those that had sham surgeries on the right hindlimb (“control rats”, *n* = 11). In additional groups, tempol (10 mg/day for 3 days) was injected into the gastrocnemius muscles of occluded rats and sham control rats. Then, 6–72 h were allowed for recovery before the experiments began.

### Examination of SOD expression

The gastrocnemius muscles from both sides were removed after the rats were anesthetized by overdose of isoflurane followed by decapitation. The red and white portions of the muscles were immediately dissected out. Western blotting analysis was used to examine the protein expression of SOD in the muscle tissues. Briefly, the concentration of protein in the homogenate was determined using a BCA reagent after the tissues were lyzed and centrifuged. SOD proteins were loaded onto gel. After electrophoresis, the proteins were electrotransferred onto polyvinylidene difluoride membranes. The membranes were then incubated with the primary antibodies: rabbit anti-SOD (1:500; Abcam). Next, the membranes were washed and incubated with an alkaline phosphatase-conjugated anti-rabbit secondary antibody (1:200; Santa Cruz). The membranes were also incubated with mouse anti-GADPH to show equal loading of the protein. The immunoreactive proteins were detected by enhanced chemiluminescence (ECL kit). The bands recognized by the primary antibody were visualized by exposure of the membrane onto an x-ray film. Then, the film was scanned and the optical density of the bands was analyzed using the Scion Image software (NIH, USA).

The immunofluorescence was also employed to examine SOD staining in muscle tissues. The red and white portions of the muscles were immediately dissected out and fixed in a 1:1 acetone and methanol solution. The tissues were then stored at −80°C and 10 μm of muscle sections were obtained using a cryostat. After being washed with PBS, the tissue were permeabilized, blocked in 0.3% Triton X-100 in PBS supplemented with 5% goat serum, and then incubated with rabbit anti-SOD antibody (1:200, Abcam) overnight at 4°C. After being washed in PBS, the sections were incubated with goat anti-rabbit Alexa Fluor 488-labeled secondary antibody (1: 200, Invitrogen) for 2 h at room temperature. Fluorescence labeled muscle tissues were examined using a Nikon Eclipse 80i microscope with appropriate filters, and the images were stored digitally on a computer.

### Examination of the exercise pressor reflex

The rats were anesthetized by inhalation of an isoflurane oxygen mixture and an endotracheal tube was inserted and attached to a ventilator. Polyethylene (PE-50) catheters were inserted into an external jugular vein and the carotid artery for saline injection and measurement of BP. PE-10 catheters were inserted into the femoral arteries for injection of drugs into the arterial blood supply of the hindlimb muscles. The skin covering the hindlimb muscles was surgically separated from the muscle below to eliminate inputs from cutaneous afferents in the hindlimb. During the experiment, end tidal CO_2_, BP, and body temperature were monitored and maintained within normal ranges (Lu et al., 2013; Xing et al., 2015).

BP was measured by connecting the carotid arterial catheter to a pressure transducer. Mean arterial pressure (MAP) was obtained by integrating the arterial signal with a time constant of 4 s. HR was determined from the arterial pressure pulse.

A laminectomy was performed to expose the lower lumbar and upper sacral portions of the spinal cord (at the L1–S1 levels) after the rats were placed in a spinal unit (Kopf Instruments) (Xing et al., 2015). The spinal roots were exposed and the right L4&5 ventral roots visually identified with assistance of an anatomical microscope. The peripheral ends of the transected L4&5 ventral roots were then placed on bipolar platinum stimulating electrodes. A pool was formed by using the skin and muscle on the back and the exposed spinal region was filled with warmed (37°C) mineral oil.

Precollicular decerebration was performed to examine the exercise pressor reflex without considering the confounding effects of anesthesia. Once the decerebration was complete, anesthesia was removed from the inhaled mixture. A recovery period of 60 min after decerebration was allowed for elimination of the effects of anesthesia from the preparation.

Static muscle contractions in the right hindlimb were performed by electrical stimulation of the L4 and L5 ventral roots (30 s, three-times motor threshold with a period of 0.1 ms at 40 Hz) in control rats (*n* = 5 without tempol; and *n* = 6 with tempol) and occluded rats (*n* = 6 without tempol; and *n* = 6 with tempol). Static muscle contraction was performed twice in each animal and 30 min were allowed between two contractions. Averaged data were used in this study.

### Statistical analysis

Experimental data were analyzed using two-way repeated measures analysis of variance (ANOVA). As appropriate, Tukey's *post-hoc* tests were used. All values were presented as mean ± SEM. For all analyses, differences were considered significant at *P* < 0.05. All statistical analyses were performed using SPSS for Windows version 15.0.

## Results

### Expression of SOD in the red and white portions of the gastrocnemius muscle

**Figure 1:**
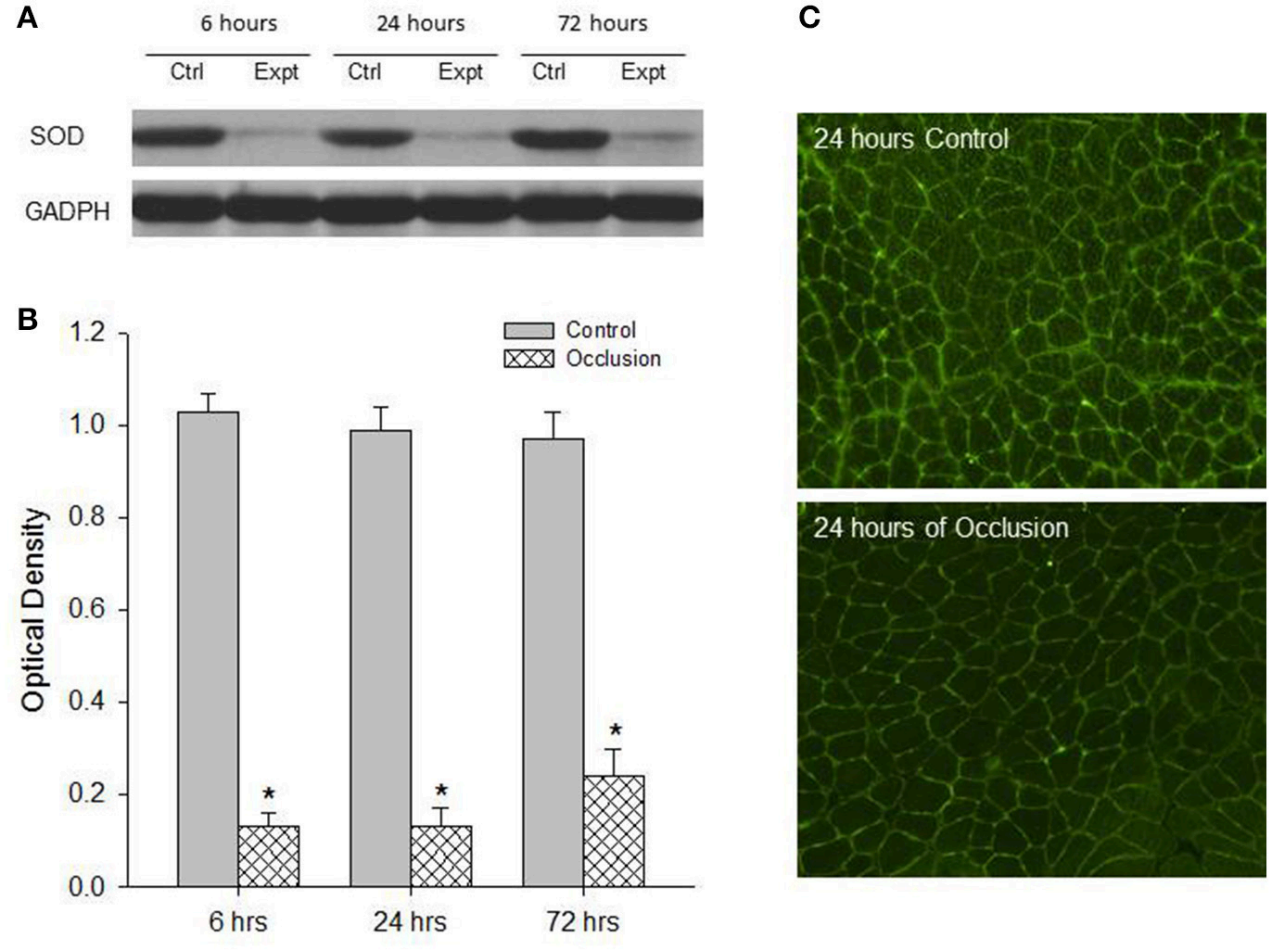
Analysis of SOD protein expression in the red muscle tissues of control limbs and limbs with 6–72 h of femoral artery occlusion. Western blot analysis was performed on muscle tissues from control limbs and occluded limbs. **(A)** Shows representative bands of SOD expression. Bands of GADPH were used as a control for equal protein loading. **(B)** Shows average data. ^*^P < 0.05 vs. control group, *n* = 6 in each group. No significant difference in SOD expression was observed among different time courses. Immunofluorescence methods were used to localize expression of SOD within the red muscles of control limbs and occluded limbs. **(C)** Typical images showing that SOD appears in the red muscles and the less fluorescence staining is observed in the muscle of occluded limbs.

**Figure 2:**
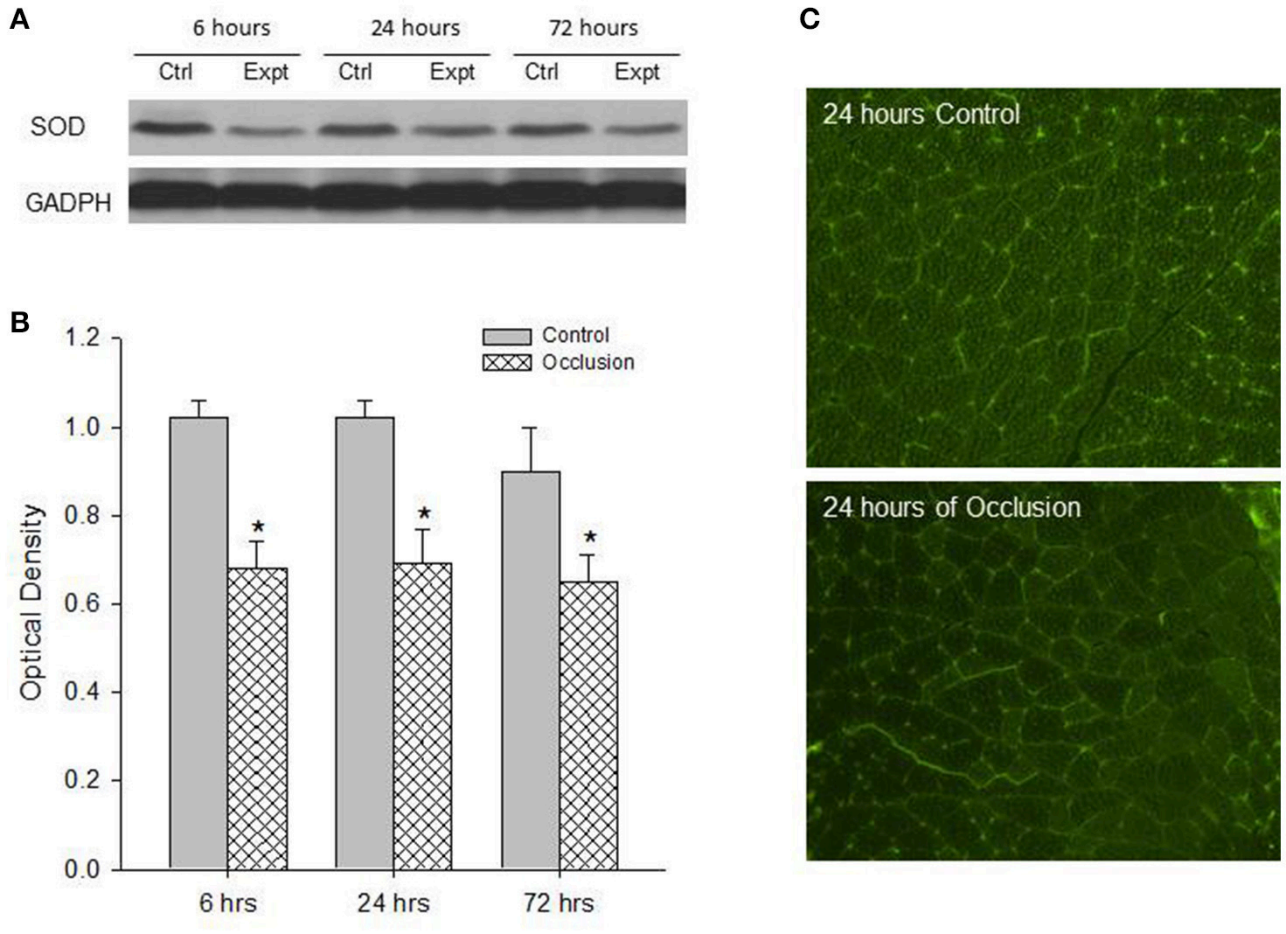
Analysis of SOD protein expression in the white muscle tissues of control limbs and limbs with 6–72 h of femoral artery occlusion. **(A,B)** Representative bands and average data of western blot analysis show that the less SOD is observed in occluded limbs. Bands of GADPH were used as a control for equal protein loading ^*^P < 0.05 vs. control group, *n* = 6 in each group. There was no significant difference in SOD expression observed among different time courses. **(C)** Typical images of immunofluorescence show that SOD is localized in the white muscles and the less fluorescence staining is observed in the muscle of occluded limbs.

First, western blot assays were performed on the red and white portions of the gastrocnemius muscle from control limbs and occluded limbs with 6, 24, and 72 h of femoral occlusion (*n* = 6 in each group). Figures 1A,B demonstrates a significant decrease observed in the levels of SOD protein within the red portion of gastrocnemius muscle of occluded limbs as compared with control limbs 6–72 h following femoral occlusion. i.e., optical density was 0.99 ± 0.05 in control vs. 0.13 ± 0.04 after 24 h of occlusion (*P* < 0.05 vs. control; *n* = 6 in each group; decreased by 87%). Similarly, Figures 2A,B shows that the levels of SOD protein were significantly decreased within the white portion of gastrocnemius muscle of occluded limbs as compared with control limbs 6–72 h following femoral occlusion. i.e., optical density was 1.02 ± 0.04 in control vs. 0.69 ± 0.08 after 24 h of occlusion (*P* < 0.05 vs. control; *n* = 6 in each group; decreased by 32%).

Second, SOD was localized within muscle tissues. The protein expression of SOD in the red and white portions of the gastrocnemius muscle of control limbs and occluded limbs (*n* = 3 in each group) was analyzed using fluorescence immunocytochemistry methods. Figure 1 illustrates immunofluorescence images (Figure 1C) that SOD was localized in the red muscle of control limbs and occluded limbs. The figure also shows that SOD staining appeared to a less degree in occluded limbs as compared with control limbs. Likewise, Figure 2 illustrates immunofluorescence images (Figure 2C) that SOD was localized in the white muscle of control limbs and occluded limbs and SOD staining appeared to a less degree in occluded limbs as compared with control limbs.

### The exercise pressor reflex following administration of tempol

**Figure 3:**
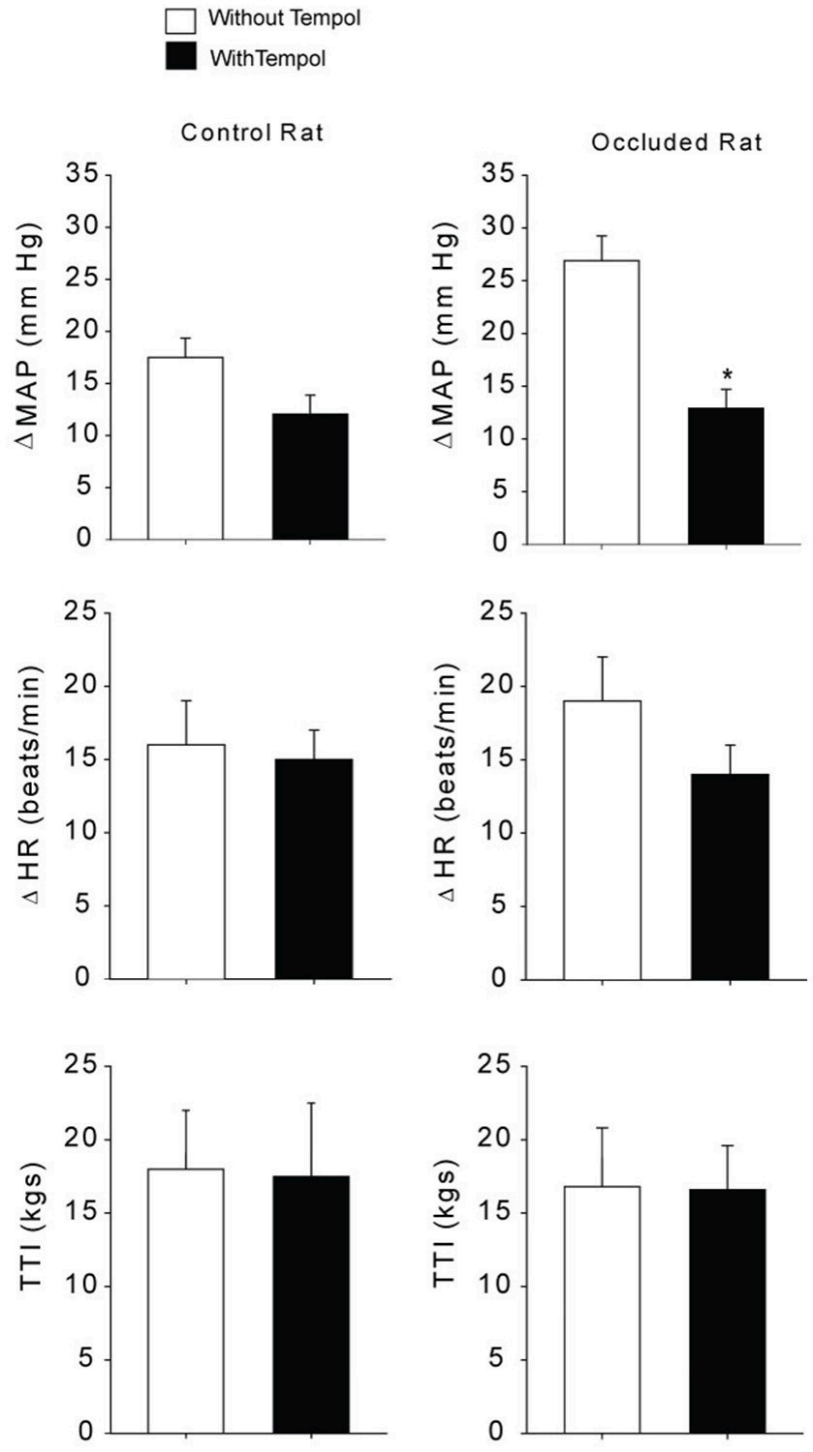
Mean arterial pressure (MAP) and heart rate (HR) responses to static muscle contraction. Without administration of tempol femoral occlusion amplified MAP response during contraction in rats with 72 h of femoral artery occlusion (*n* = 6) as compared with control rats (*n* = 5). **Left**, previous administration of tempol failed to alter MAP response in control rats (*n* = 6). **Right**, previous administration of tempol significantly attenuated the MAP response in occluded rats (*n* = 6) as compared with occluded rats without tempol (*n* = 6). ^*^P < 0.05 vs. occluded rats with no tempol. Note that there was no significant difference in developed muscle tension, expressed as time-tension index (TTI).

Baseline values for MAP and HR before static contraction had no significant differences in control rats (*n* = 5) and occluded rats (*n* = 6). Basal MAP and HR were 98 ± 12 mmHg and 390 ± 15 beats/min in control rats; and 95 ± 15 mmHg, and 387 ± 18 beats/min in occluded rats (*P* > 0.05 vs. controls for MAP and HR). Tempol did not alter basal MAP and HR in control rats and occluded rats (*n* = 6 in each group). After tempol injection, they were 92 ± 16 mmHg and 382 ± 22 beats/min in control rats; and 97 ± 13 mmHg and 395 ± 20 beats/min in occluded rats (*P* > 0.05 vs. respective groups without tempol for MAP and HR). Figure 3 shows MAP responses to muscle contraction after administration of tempol in control rats and occluded rats. As compared with control rats, femoral occlusion significantly increased MAP response (*P* < 0.05 vs. controls). Tempol attenuated increases of MAP response observed in occluded rats. In the occluded rats, MAP and HR responses were 26 ± 3 mmHg and 19 ± 3 bpm with no tempol (*n* = 6); and 12 ± 2 mmHg, and 14 ± 2 bpm with tempol application (*P* < 0.05 vs. occluded group with no tempol for MAP response; *n* = 6). Tempol failed to attenuate MAP and HR responses in control rats. The responses were 18 ± 2 mmHg and 16 ± 3 bpm with no tempol (*n* = 6); and 12 ± 2 mmHg, 15 ± 2 bpm with tempol application (*P* > 0.05, tempol group vs. group with no tempol). It is noted that there were no differences observed in muscle tension development (expressed as time-tension index, TTI) among groups (*P* > 0.05). In control rats, TTIs were 13.5 ± 0.5 kgs with no tempol and 11.8 ± 1.2 kgs with tempol. In occluded rats, TTIs were 12.1 ± 1.2 kgs with no tempol and 13.5 ± 1.1 kgs with tempol.

## Discussion

The new findings from this study include that (1) femoral artery occlusion impaired SOD in the oxidative (red) and glycolytic (white) muscles of the hindlimb; and (2) a chronic compensation of SOD by local injections of tempol attenuated the amplified exercise pressor reflex following femoral occlusion.

The effects of ROS on the exercise pressor reflex in experimental PAD rats have been studied. A bolus injection of tempol into arterial blood supply of the hindlimb muscles attenuated the exercise pressor reflex in occluded rats (McCord et al., 2011). In this prior study, tiron was not observed to have the inhibitory effects on the exercise pressor reflex in either freely perfused rats or occluded rats when was given in the same way (McCord et al., 2011). It is noted that in this prior study tempol or tiron was trapped in the muscles and ~10–15 min later the hindlimb muscle was reperfused and contracted. However, tiron was shown to attenuate the exercise pressor reflex in occluded rats when it was constantly infused into the hindlimbs while the hindlimb muscles were contracting (Harms et al., 2017). Infusion of tiron also attenuated muscle activity of NADPH oxidase (Harms et al., 2017). The method and period to deliver the antioxidants was likely to lead to the different effects of the antioxidants on the reflex. Consistent with the previous findings, data of the current study showed that oxidative stress is engaged in the amplified exercise pressor reflex induced by femoral occlusion and antioxidants can improve the exaggeration in the exercise pressor reflex.

It should be noted that in those previous studies tempol and/or tiron were given into the muscle tissues for less than an hour and then the reflex was evoked (McCord et al., 2011; Harms et al., 2017). However, in our current study, we administered tempol into the occluded muscle for 3 days before the reflex was evoked. This approach allowed chronic compensating effects of SOD in occluded muscles because muscle SOD was impaired by femoral occlusion. Our data demonstrated that chronic administration of tempol into the muscles of rats in which SOD was impaired by femoral occlusion attenuated amplification of the exercise pressor reflex.

In addition, a prior study observed that tempol infused into the femoral artery of the contracted muscles attenuated the exercise pressor reflex in healthy rats and in those with heart failure (Wang et al., 2009). In contrast, the similar dosage of tempol was reported to attenuate the exercise pressor reflex in rats with heart failure, but not in healthy rats (Koba et al., 2009). Intravenous infusion of ascorbic acid, an antioxidant, had no effect on the reflex in healthy humans but attenuated the reflex in PAD patients (Muller et al., 2012). Taken together with all those findings, there is a general agreement that ROS plays a role in modulating the exercise pressor reflex in the diseased conditions such as PAD and heart failure.

The effect of fiber-type composition on the pressor reflex evoked by static muscle contraction was studied in cats. Static contraction of the soleus muscle (slow twitch oxidative) induced no increase in BP; whereas contraction of the medial gastrocnemius muscle (mixed fiber type) caused an increase in BP (Petrofsky and Lind, 1980). However, an increase in BP was observed from red muscle although it was smaller than that from white muscles (Iwamoto and Botterman, 1985). Moreover, static contraction of a predominately glycolytic muscle evoked a larger pressor response as compared with that elicited by contraction of a primarily oxidative muscle (Wilson et al., 1995). Thus, muscle fiber type has an effect on the magnitude of the sensory afferent feedback during exercise. Our prior study further examined responsiveness of sensory neurons innervating muscles comprised predominately of the oxidative and glycolytic fibers to protons and capsaicin (Xing et al., 2008), demonstrating that amplitude of inward currents responsive to protons and capsaicin in neurons innervating glycolytic muscle is greater than that innervating oxidative muscle.

In the current study, we examined the levels of SOD in oxidative and glycolytic muscles of the hindlimb following femoral artery occlusion. In both phenotypes of muscles femoral occlusion downregulated SOD and it is noted that this effect appeared to a greater degree in oxidative muscles. It has been reported that oxidative and glycolytic muscles are largely protected against the deleterious effects of ischemia reperfusion when previously treated with tempol. The activity of antioxidant enzymes, particularly glutathione peroxidase (GPx), is lower in glycolytic than oxidative muscles (Picard et al., 2012). Nevertheless, impaired SOD in occluded muscles is likely to affect the exercise pressor reflex and a chronic compensation of SOD by a local administration of tempol into the muscles can improve the exaggerated reflex evoked by femoral occlusion.

### Study limitations

Three forms of SOD are present in humans and in all other mammals (Perry et al., 2010). SOD1 is located in the cytoplasm, SOD2 is in the mitochondria, and SOD3 is extracellular. In our current study, we did not determine which form of SOD was expressed in the skeletal muscles. Nonetheless, results of our immunohistological experiment show that SOD was likely extracellular and thus it is speculated that SOD3 was altered after the femoral artery occlusion in the skeletal muscle and engaged in the amplified exercise pressor reflex. In addition, tempol scavenges superoxide and can generate more H_2_O_2_. An increase in ROS production, predominantly HO^·^ and H_2_O_2_, has been observed in different contraction protocols (O'Neill et al., 1996; Palomero et al., 2008). H_2_O_2_ is a vasodilator (Breton-Romero and Lamas, 2014) and we did not examine the levels of H_2_O_2_ in the skeletal muscles, Thus, another study limitation is that we were not clear if the lowering effect of tempol on BP during static exercise was directly related to H_2_O_2_-induced vasodilation or somehow was via other complex signaling mechanisms initiated by removal of oxidative stress.

In conclusion, SOD is impaired in the oxidative and glycolytic muscles following femoral occlusion and chronic supplementation of SOD in ischemic muscles has inhibitory effects on the amplified exercise pressor reflex, which is likely to improve the restricted blood flow to the limbs in PAD.

## Author contributions

JX, and JLi: participated in the design of the experiment and drafting the manuscripts; JX, JLu, and JLiu: contributed to the collection and analysis; JLi: contributed to revising the article critically for important intellectual content; All authors approved the final version.

### Conflict of interest statement

The authors declare that the research was conducted in the absence of any commercial or financial relationships that could be construed as a potential conflict of interest.
